# Recurrent upper lumbar disc herniation treated via the transforaminal approach using microendoscopy-assisted lumbar discectomy: a case report

**DOI:** 10.1186/s13256-018-1653-8

**Published:** 2018-04-27

**Authors:** Yasutaka Takagi, Hiroshi Yamada, Hidehumi Ebara, Hiroyuki Hayashi, Satoshi Kidani, Kazu Toyooka, Yoshiyuki Kitano, Kenji Kagechika, Hiroyuki Tsuchiya

**Affiliations:** 1 Department of Orthopaedic Surgery, Tonami General Hospital, 1-61 Shintomi-cho, Tonami City, Toyama 939-1395 Japan; 2 Department of Rehabilitation Medicine, Kanazawa Medical University, 1-1 Daigaku, Uchinada-machi, Kahoku-gun, Ishikawa 920-0293 Japan; 3 Department of Orthopaedic Surgery, Graduate School of Medicine, Kanazawa University, 13-1 Takara-machi, Kanazawa City, Ishikawa 920-8641 Japan

**Keywords:** Microendoscopy-assisted lumbar discectomy (MED), Recurrent upper lumbar disc herniation, Transforaminal approach, Percutaneous endoscopic lumbar discectomy (PELD)

## Abstract

**Background:**

Although microendoscopy-assisted lumbar discectomy for lateral or extraforaminal lumbar disc herniations via the lateral approach has previously been reported, microendoscopy-assisted lumbar discectomy for central or paramedian disc herniations via the lateral approach has not been reported.

We report the first case of recurrent upper lumbar disc herniation (L2–L3) treated with microendoscopy-assisted lumbar discectomy via the transforaminal approach. No microendoscopy-assisted lumbar discectomy for recurrent upper lumbar disc herniation via the transforaminal approach has previously been reported. Percutaneous endoscopic lumbar discectomy via the transforaminal approach is very useful as a minimally invasive surgery for disc herniations. We applied percutaneous endoscopic lumbar discectomy via the transforaminal approach, and invented a new microendoscopy-assisted lumbar discectomy via the transforaminal approach.

**Case presentation:**

A 79-year-old Japanese man was operatively managed for recurrent L2–L3 herniation. An 18 mm skin incision was made approximately 70 mm from the midline to the lateral side to allow a sufficiently angled trajectory to the extraforaminal space. The transforaminal approach was used. The exiting nerve root was identified along its course inferior to the pedicle. The lateral portion of the pars interarticularis and the facet joint was removed using a high-speed drill under the guidance of an endoscope. The tip of the endoscope was set at the lateral side of the dura mater. The dura mater was retracted medially and gently, and the herniated disc fragments were removed safely. All symptoms were relieved postoperatively. Postoperative magnetic resonance imaging demonstrated disappearance of all herniated disc fragments. A postoperative three-dimensional computed tomographic scan demonstrated the complete preservation of the facet joint.

**Conclusions:**

This is the first report of a case of recurrent upper lumbar disc herniation treated with microendoscopy-assisted lumbar discectomy via the transforaminal approach. This procedure allows for the use of a nerve retractor and other instruments to detach adhesions from the dura mater. This procedure has the advantages of clear visualization of the dura mater, exiting nerve root, and traversing nerve root, and diminished risk of nerve injury, and complete preservation of the articular surface of the facet joint.

## Background

Although microendoscopy-assisted lumbar discectomy (MED) for lateral or extraforaminal lumbar disc herniations via the lateral approach has previously been reported, MED for central or paramedian disc herniations via the lateral approach has not been reported.

Percutaneous endoscopic lumbar discectomy (PELD) via the transforaminal approach is very useful as a minimally invasive surgery for disc herniations. We applied PELD via the transforaminal approach, and invented a new MED via the transforaminal approach.

We treated a case of recurrent upper lumbar disc herniation using MED via the transforaminal approach. All procedures were performed safely with endoscopic assistance.

## Case presentation

**Fig. 1:**
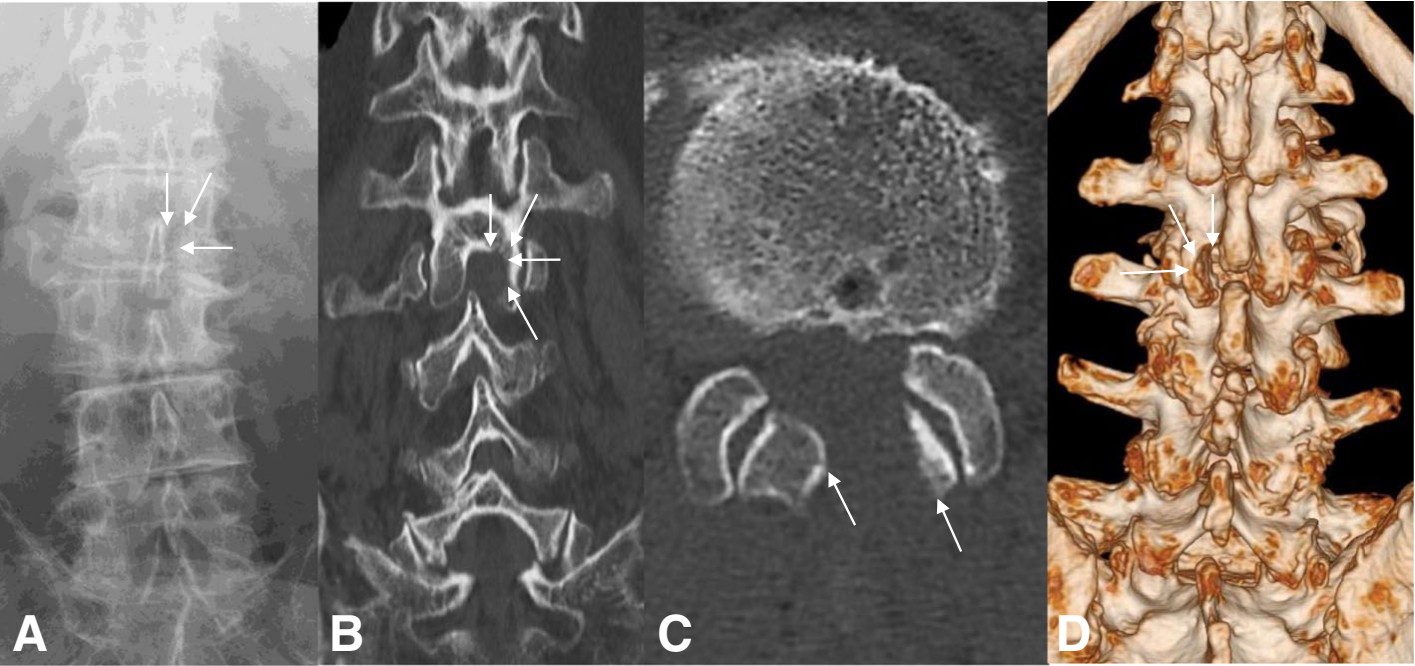
X-ray, computed tomography, and reconstruction three-dimensional computed tomography shows the interlaminar window open and the facet joint preserved. **a** X-ray. **b** Coronal view. **c** Axial view. **d** Three-dimensional computed tomography. The *arrows* are pointing to the interlaminar window

**Fig. 2:**
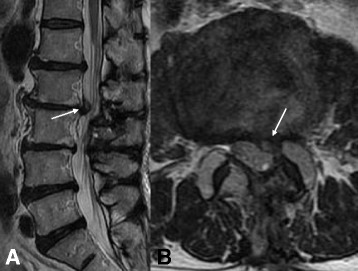
Magnetic resonance imaging shows recurrent upper lumbar disc herniation at the L2–L3 level. **a** Sagittal view. **b** Axial view. The *arrows* are pointing to recurrent upper lumbar disc herniation at the L2/3 level

A 79-year-old Japanese man presented with a 5-month history of radicular pain in his left gluteal region and his lateral thigh. He was unemployed and had no relevant family history, and no history of tobacco smoking and alcohol consumption. Initial management consisted of pharmacologic pain control and selective root block. However, 1 month later, his pain had increased, and he had undergone an operation for lumbar disc herniation at the L2–L3 level using MED. He experienced immediate pain relief after the surgery. Two months later, his leg pain reappeared. Computed tomography (CT) and reconstruction three-dimensional (3D) CT showed that the interlaminar window was open and the facet joint was preserved (Fig. 1). Magnetic resonance imaging (MRI) showed recurrent upper lumbar disc herniation at the L2–L3 level (Fig. 2). Since caudal block and selective lumbar nerve block were effective for a short period, surgery was planned. A neurological examination showed muscle weakness of his left quadriceps femoris muscles (power, 4 out of 5) and decreased sensation in his left lateral thigh. Laboratory assessments showed no sign of inflammatory reaction: C-reactive protein 0.02 mg/L, white blood cell count 6.5 × 10^9^/L, and platelet count 134 × 10^9^/L. In addition, a laboratory assessment of liver and renal function showed no abnormal findings: aspartate aminotransferase (AST) 16 U/L, alanine aminotransferase (ALT) 15 U/L, alkaline phosphatase (ALP) 256 U/L, blood urea nitrogen (BUN) 12.2 mg/dl, and creatinine 0.44 mg/dl. Urine analysis, serology, and microbiology showed no abnormal findings. His body temperature was 35.6 degrees, pulse was 67, and blood pressure was 126/62 mm/Hg at the time of admission.

### Surgical technique

**Fig. 3:**
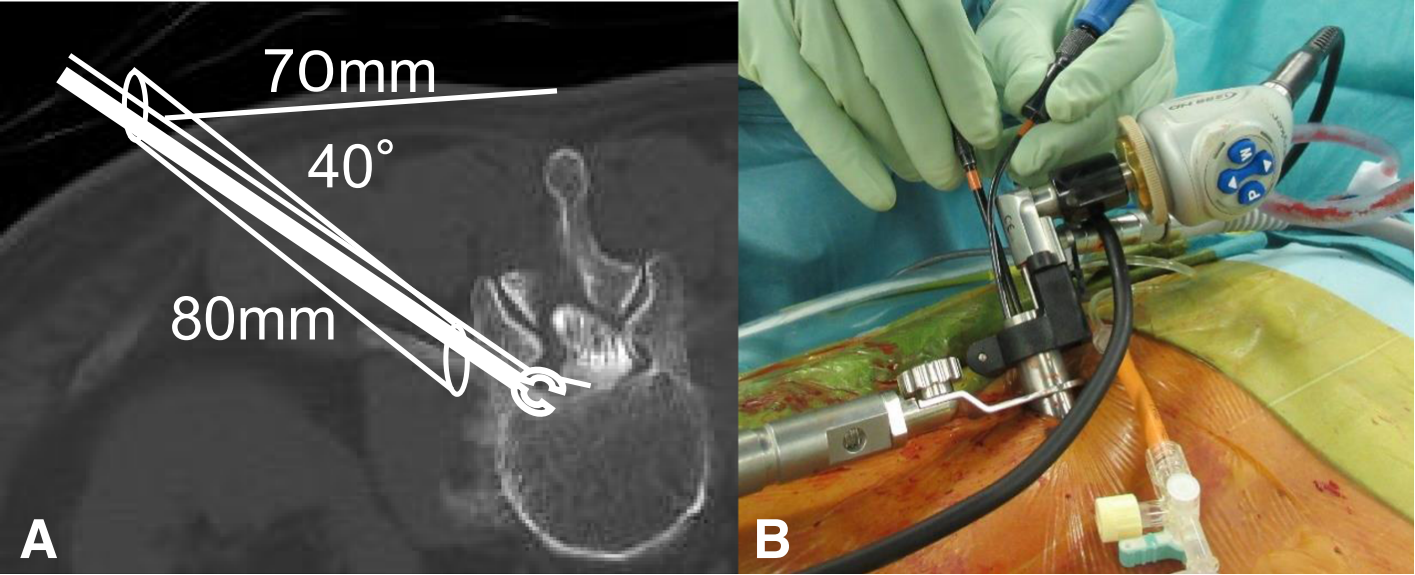
**a** Operation plan on computed tomography myelogram and **b** operation view

**Fig. 4:**
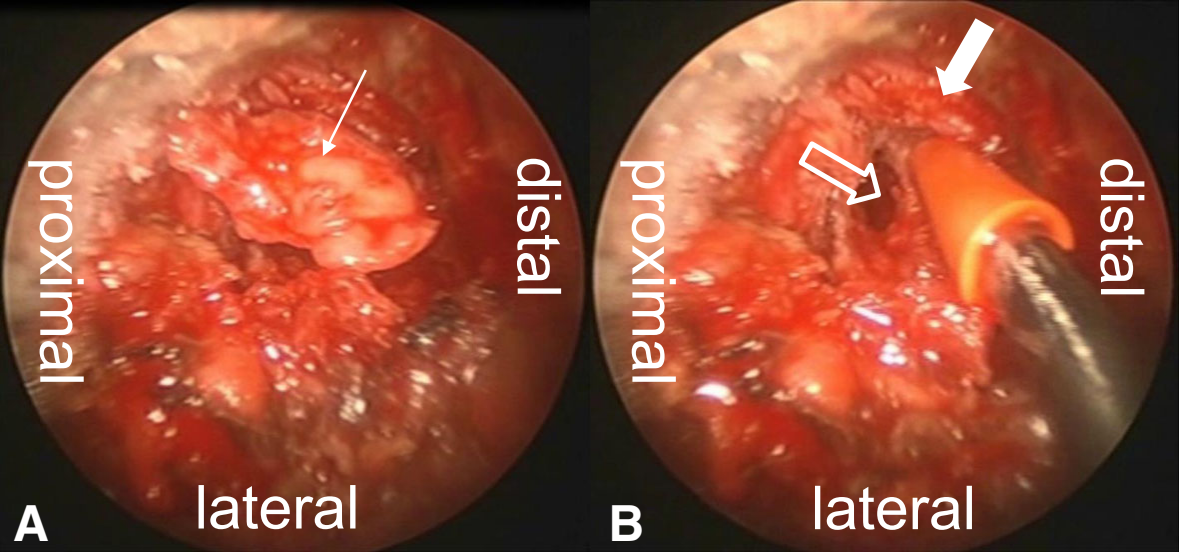
Endoscopic views. **a** *Arrow* is disc herniation. **b** *White outline arrow* is L2–L3 disc space and *white solid arrow* is traversing nerve root

The L2–L3 level was localized using intraoperative fluoroscopy, and an 18 mm transverse skin incision was made approximately 70 mm from the midline to the left side to allow a sufficiently angled trajectory to the L2–L3 extraforaminal space. Then, a 16 mm tubular retractor was positioned, and the endoscope was placed within the tube (Fig. 3). The soft tissue overlying the lateral facet and left L2 transverse process was dissected, and a high-speed drill was then used to remove the inferior portion of the left L2 transverse process and shave down small portions of the lateral facet and the inferolateral portion of the left L2 pars interarticularis. Careful blunt dissection allowed for the identification of the left L2 nerve root (exiting nerve root) along its course, inferior to the pedicle of L2. The lateral portion of the pars interarticularis and the facet joint was removed using a high-speed drill under the guidance of the endoscope. The yellow ligament and adhesive tissues were removed, and the dura mater was revealed. The top of the camera lens lay over the tubular retractor, and it was possible to see the dura mater and the exiting nerve root just from the lateral side of the thecal sac, using a 25 degree endoscope. Gentle retraction of the dura mater medially allowed for exposure of the L2–L3 recurrent disc herniation. The herniated disc fragments were detached from the dura mater and the left L3 nerve root (traversing nerve root) safely using a nerve root retractor. The herniated disc fragments were removed safely. Then, L2–L3 annulotomy and routine disc removal were performed, and the dura mater and traversing nerve root were seen to be relaxed and well decompressed (Fig. 4).

**Fig. 5:**
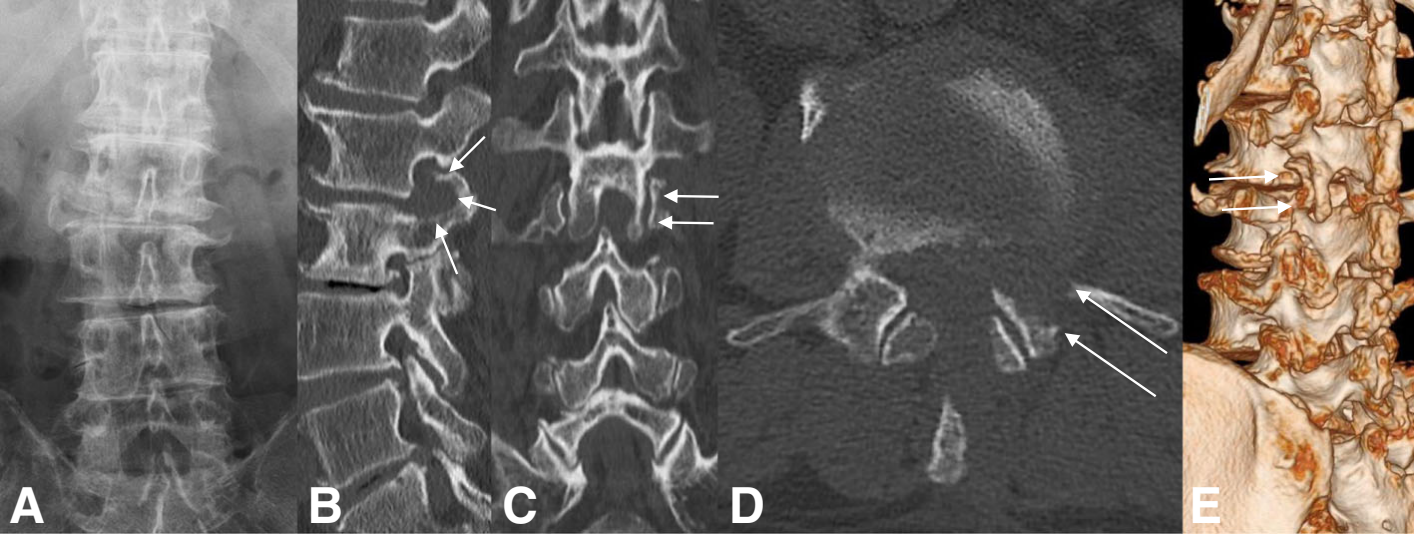
Postoperative X-ray and computed tomography demonstrated the complete preservation of the articular surface of the facet joint. **a** X-ray; **b** sagittal view; **c** coronal view; **d** axial view; and **e** three-dimensional computed tomography – oblique view. The *arrows* are pointing to the the complete preservation of the articular surface of the facet joint and the trajectory to the L2-3 extraforaminal space

**Fig. 6:**
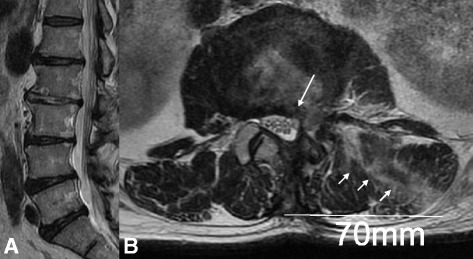
Magnetic resonance imaging demonstrated that all herniated disc fragments had disappeared. **a** Sagittal view and **b** axial view. The trajectory of the operation is demonstrated (*short white arrow*). All herniated disc fragments had disappeared (*long white arrow*)

Our patient experienced immediate pain relief after the surgery. Postoperative X-ray and CT demonstrated the complete preservation of the articular surface of the facet joint (Fig. 5). Postoperative MRI demonstrated that all herniated disc fragments had disappeared and the trajectory to the L2–L3 extraforaminal space was demonstrated (Fig. 6). No surgery-related complications, such as dural laceration, nerve root injury, hematoma, or infection, were encountered. At the 12-month follow-up, his leg pain had been relieved and no signs of sciatica were present.

## Discussion

Surgical outcomes for patients with disc herniation at the upper lumbar level (L1–L2 and L2–L3) were less satisfactory than for those treated at lower lumbar levels. Sanderson *et al*. reported that the surgical outcome, regarding postoperative back and radicular pain, is worse for herniated discs at L1–L2 and L2–L3, as compared with those at L3–L4 [1]. Gutterman and Shenkin reported that patients with L2–L3 disc herniation had a 53% success rate, as compared with 83% for patients with L3–L4 disc herniation [2]. The anatomic characteristics of the upper lumbar spine are: (1) the distance between the two pars interarticularis is narrow, therefore, even the shortest lateral deviation during a laminotomy could result in the loss of the inferior facet and subsequent instability; (2) the distance between the upper and lower margins of the lamina is greater; (3) the interlaminar window is narrow and the inferior border of the lamina overhangs more of the disc space, which is further compounded by the fact that upper lumbar disc herniation usually occurs in older patients whose height has already decreased owing to disc degeneration; and (4) the diameter of the thecal sac at the upper lumbar level is larger than that at the lower lumbar region. Wide laminectomy is needed to expose the disc space because of the narrow distance between the two pars interarticularis, whereas trying to prevent neural tissue retraction could lead to the removal of the whole facet and segmental instability [3].

Since the introduction of the concept of percutaneous posterolateral nucleotomy by Kambin and Zhou in 1973, the technique of PELD has evolved over the years and is increasingly becoming a preferred choice of treatment for lumbar disc herniation [4]. Wu *et al*. reported that of the 12 patients in a PELD at L1–L2 and L2–L3 group, four exhibited excellent, six had good, one had fair, and one had poor outcomes, according to Macnab criteria assessment [5].

We must consider scar tissue and fibrosis in recurrent radicular pain after discectomy. The transforaminal approach in PELD clearly bypasses the previous dorsal part of the scar tissue and reduces the risk for dural tear.

Use of the conventional posterior approach to an upper lumbar disc herniation may sometimes increase the risk of damage to the spinal cord or the exiting nerve root, due to an insufficient operative field caused by the narrow lamina window of the upper lumbar spine [6]. To avoid these issues, we invented a new MED via the transforaminal approach. Endoscope-assisted transtubular surgery, recently called MED, was spread by the efforts of Destandeau, as well as by Foley and Smith [7, 8]. Recently, this technique has also been applied to the extraforaminal zone. It allows for minimally invasive visualization of the site of the lesion, regardless of its depth. However, no MED for central or paramedian disc herniations via the lateral approach has been reported. In addition, no MED for recurrent upper disc herniation (L2–L3) via the transforaminal approach has previously been reported.

Kim *et al*. reported on the oblique paraspinal approach, which utilizes an operating microscope in the upper lumbar herniation and thoracolumbar junction [9]. A 30 to 40 mm longitudinal skin incision was made approximately 30 mm lateral from the midline. The lateral portion of the pars interarticularis and facet join was removed.

Although MED for lateral or extraforaminal lumbar disc herniations via the lateral approach has previously been reported, MED for central or paramedian disc herniations via the lateral approach has not been reported. In the present case, we applied PELD via the transforaminal approach and invented a new MED for paramedian disc herniation via the transforaminal approach. This new MED via the transforaminal approach can completely preserve the articular surface of the facet joint.

In this procedure, an 18 mm transverse skin incision was made approximately 70 mm from the midline to the lateral side. This procedure allows for the lateral aspect of the dura mater and the exiting nerve root to be seen. The herniated disc fragments were safely detached from the dura mater by a nerve root retractor and other instruments. All herniated disc fragments were removed safely, and the dura mater and traversing nerve root were seen to be relaxed and well decompressed. The articular surface of the facet joint was completely preserved. The entire procedure was performed safely with endoscopic assistance.

## Conclusions

This is the first report of a case of recurrent upper lumbar disc herniation treated with MED via the transforaminal approach. This procedure allows for the use of a nerve retractor and other instruments to detach adhesions from the dura mater. This procedure has the advantages of clear visualization of the dura mater, exiting nerve root, and traversing nerve root, and diminished risk of nerve injury, and complete preservation of the articular surface of the facet joint.

## Abbreviations

CT: Computed tomography
MED: Microendoscopy-assisted lumbar discectomy
MRI: Magnetic resonance imaging
PELD: Percutaneous endoscopic lumbar discectomy
